# *Lycium barbarum* Polysaccharide Mediated the Antidiabetic and Antinephritic Effects in Diet-Streptozotocin-Induced Diabetic Sprague Dawley Rats via Regulation of NF-*κ*B

**DOI:** 10.1155/2016/3140290

**Published:** 2016-04-21

**Authors:** Mingzhao Du, Xinyu Hu, Ling Kou, Baohai Zhang, Chaopu Zhang

**Affiliations:** ^1^Affiliated Hospital of Jiangsu University, Jiangsu University, Zhenjiang 212001, China; ^2^Faculty of Medicine, Changchun Medical College, Changchun 130013, China

## Abstract

*Lycium barbarum*, extensively utilized as a medicinal plant in China for years, exhibits antitumor, immunoregulative, hepatoprotective, and neuroprotective properties. The present study aims to investigate the hyperglycemic and antidiabetic nephritic effects of polysaccharide which is separated from* Lycium barbarum* (LBPS) in high-fat diet-streptozotocin- (STZ-) induced rat models. The reduced bodyweight and enhanced blood glucose concentration in serum were observed in diabetic rats, and they were significantly normalized to the healthy level by 100 mg/kg of metformin (Met) and LBPS at doses of 100, 250, and 500 mg/kg. LBPS inhibited albuminuria and blood urea nitrogen concentration and serum levels of inflammatory factors including IL-2, IL-6, TNF-*α*, IFN-*α*, MCP-1, and ICAM-1 compared with diabetic rats, and it indicates the protection on renal damage. Furthermore, the activities of SOD and GSH-Px in serum were enhanced strikingly by LBPS which suggests its antioxidation effects. LBPS, compared with nontreated diabetic rats, inhibited the expression of phosphor-nuclear factors kappa B (NF-*κ*B) and inhibitor kappa B alpha in kidney tissues. Collectively, LBPS possesses antidiabetic and antinephritic effects related to NF-*κ*B-mediated antioxidant and antiinflammatory activities.

## 1. Introduction

As reported, nearly 2.2% of total death worldwide is caused by diabetes mellitus [1], which is characterized by hyperglycemia and metabolic disturbance including lipids, carbohydrates, and proteins [2]. Type II diabetes, the most common form of diabetes, is related to insulin resistance, and it will affect over 8 billion people till 2025 worldwide [3]. Combining with insulin secretion deficiency, hyperlevel of blood glucose is observed in diabetic patients; what is worse, several complications, such as nephropathy, endanger the patients' lives [3]. Diabetic nephropathy is the major cause of end-stage renal disease, and various pathways are involved during this process [4]. It has been confirmed that the reduction of oxidative stress and the enhancement of host antioxidant defense system are related to diabetic nephropathy therapy [5]. The levels of superoxide dismutase (SOD) are related to human aging and health; meanwhile, glutathione peroxidase (GSH-Px) displays protective effects on cell structure and membrane function [6]. The inflammatory cytokines including interleukin play central roles during diabetic nephropathy attack and treatment [7].

Till now, the traditional treatments only focus on regulating blood glucose levels, which fail to control complications [8]. Insulin injection and some oral antihyperglycemic agents, such as metformin, display undesirable side effects including insulin resistance, hypoglycemia, and gastrointestinal disturbances [9]. Due to the limitation of existing antidiabetic agents, a search for alternative treatment is highly demanded.

As reported previously, natural products turn out to be a valuable reservoir for searching novel drugs [10]. Scientific evidence has been collected to support* Rhizoma coptidis* being used as an effective agent for the treatment of diabetes mellitus [11]. Danhong injection successfully inhibits diabetic retinopathy and nephropathy via ameliorating glucose metabolism [12].* Lycium barbarum*, extensively utilized as a medicinal plant in China, exhibits immune enhancing, hepatoprotective, and neuroprotective properties [13]. As one of important bioactive components,* Lycium barbarum* polysaccharide (LBPS) prevents 6-OHDA-caused PC12 cell apoptosis partially via reactive oxygen species- (ROS-) nitric oxide (NO) pathway [14]. In both* in vitro* and* in vivo* experiments, LBPS shows synergistic immunotherapeutic effects with interferon-*α*2b on renal cell carcinoma [15]. In high-fat diet-induced insulin resistance animal models, LBPS is confirmed to be an antioxidant against insulin resistance via activating phosphatidyl inositol 3-kinase (PI3K)/AKT/nuclear factor erythroid 2 p45 related factor 2 (Nrf2) pathway. Furthermore, LBPS increases insulinogenic index and high density lipoprotein (HDL) levels in patients with type II diabetes [16]. Purified LBPS has significantly inhibited the absorption of glucose in a dose-dependent manner in cell experiments [17]. These encouraging data let us further investigate the antidiabetic and antinephritic effects of LBPS and underlying mechanism systematically.

The present study aims to investigate the hyperglycemic and anti-inflammatory effects of LBPS in high-fat diet-streptozotocin- (STZ-) induced rat models. During the experiments, serum indexes were measured to verify the positive effects of LBPS; meanwhile the activations of nuclear factors kappa B (NF-*κ*B) in kidney were detected via western blot to analyze its possible mechanisms.

## 2. Materials and Methods

### 2.1. *Lycium barbarum* Polysaccharide Preparation

100 g of* Lycium barbarum* (obtained from pharmacy in Affiliated Hospital of Jiangsu University, Jiangsu, China) was extracted two times in 500 mL hot water at 80°C for 3 h. After centrifuging, the supernatant was sequentially concentrated, and the existing proteins were removed using Sevag reagent [V (n-butanol) : V (chloroform) = 1 : 4, 50 mL]. After adding fourfold ethanol, the supernate was placed at 4°C overnight. The precipitate polysaccharide (LBPS) was collected and freeze-dried for further experiments.

### 2.2. Diet-STZ-Induced Diabetic Rat Model Establishment

The experimental animal protocol was approved by the Animal Ethics Committee of Jiangsu University. Sprague Dawley rats (male; 8 weeks; 180–220 g) were housed under the standard laboratory conditions of 22°C ± 1°C, relative humidity of 55%, and 12-h : 12-h light/dark cycle (lights on 7:00–19:00 h) during the study. The animals were given standard rat pellets and tap water* ad libitum*.

Rats (*n* = 8) fed with the standard control diet following with normal saline injection served as control group. Another 40 rats were fed with a high-fat diet (HFHSD, 12% protein, 5% fat, 67% carbohydrate, 5% cholesterol, and 5% other additives) for 8 weeks and then injected with 30 mg/kg streptozotocin (STZ; Sigma, USA) dissolved in a citrate buffer (0.1 mol/L sodium citrate and 0.1 mol/L citric acid, pH 4.5) for one week. After the last STZ injection, the blood glucose level was detected, and rats with the value of more than 11.1 mmol/L were defined as with diabetes.

### 2.3. Animal Treatment Process

Rats (*n* = 8) in control group were given 2.0 mL/kg sterile saline for four weeks. Diabetic rats were divided into five groups randomly and orally treated with 2.0 mL/kg sterile saline (model rats, *n* = 8), 100 mg/kg of metformin hydrochloride (Met; Sigma, USA) (positive control rats, *n* = 8), LBPS at doses of 100 mg/kg (low dose-treated rats, *n* = 8), 250 mg/kg (middle dose-treated rats, *n* = 8), and 500 mg/kg (high dose-treated rats, *n* = 8). The oral administration lasted for four weeks, and the bodyweight and blood glucose level of each rat were recorded every week.

### 2.4. Oral Glucose Tolerance Test (OGTT) in Rats

OGTT was performed to avoid a false positive result which is obtained from the monitor of blood glucose. The experimental rats were fasted for 12 h after the last administration of LBPS and Met and then orally treated with 2.0 g/kg glucose. The blood glucose levels were analyzed, respectively, at 0, 30, 60, 90, and 120 min. The area under the blood glucose curve (AUC) was calculated [18] according to

(1) AUC=basal  glycaemia+glycaemia  0.5 h×0.25+glycaemia  0.5 h+glycaemia  1 h×0.25+glycaemia  1 h+glycaemia  2 h×0.5.

### 2.5. Blood Biochemical Indexes Analysis

At the end of the experiment, blood samples were collected from the caudal vein of each rat. The serum levels of insulin (INS), interleukin-2 (IL-2), interleukin-6 (IL-6), tumor necrosis factor-*α* (TNF-*α*), interferon-*α* (IFN-*α*), monocyte chemoattractant protein 1 (MCP-1), and intercellular adhesion molecule (ICAM-1) were detected by using enzyme-linked immunosorbent assay (ELISA) kits according to operation manual (Calbiotech, USA). The activities of SOD and GSH-Px in serum and the levels of blood urea nitrogen (BUN) and albuminuria in urine were analyzed via commercial kits obtained from Nanjing Biotechnology Co. Ltd. (Nanjing, China).

### 2.6. Histopathological Examination

Collected kidney tissue was immersed in 4% paraformaldehyde for 48 h and then dehydrated step by step using 50–100% ethanol. Samples were immersed in xylene for 30 min and incubated in paraffin at 65°C overnight. Once embedded in wax, samples were cut serially into 5 *μ*m thick sections using a microtome (Leica, Germany) and spread over microscopy slides. The sections were deparaffinized with fresh xylene for 10 min, rehydrated with a gradient of ethanol (100%, 90%, 80%, and 70%), washed three times with DD water, analyzed via hematoxylin and eosin (H&E) staining, and photographed with a light microscope digital camera (Nikon Instruments, Tokyo, Japan).

### 2.7. Western Blot

Collected kidney tissues were homogenized in radioimmunoprecipitation assay buffer (RIPA; Sigma-Aldrich, USA) which contains 1% protease inhibitor cocktail (Sigma-Aldrich, USA) and 2% phenylmethanesulfonyl fluoride (PMSF; Sigma-Aldrich, USA). Protein concentration was analyzed via Bradford method, and 30 *µ*g samples were separated by using a 10% SDS-PAGE gel and transferred electrophoretically onto nitrocellulose membranes (0.45 m; Bio Basic, Inc., USA). After being blocked in 5% bull serum albumin (BSA) for 4 h at 4°C, the membranes were blotted at 4°C overnight with primary antibodies phosphor-nuclear factor-*κ*B (P-NF-*κ*B) receptor, total-NF-*κ*B (T-NF-*κ*B) (1 : 500; Santa Cruz, USA), inhibitor kappa B alpha (I*κ*B*α*), and glyceraldehyde-3-phosphate dehydrogenase (GAPDH) (Santa Cruz, USA) at dilution of 1 : 500. The membranes were further incubated with horseradish peroxidase-conjugated secondary antibodies (1 : 2000; Santa Cruz, USA). Chemiluminescence was detected by using ECL detection kits (GE Healthcare, UK). The intensity of the bands was quantified by scanning densitometry using software Image J (National Institutes of Health, Bethesda, USA).

### 2.8. Statistical Analysis

All values were expressed as mean ± SD. A one-way analysis of variance (ANOVA) was used to detect statistical significance followed by* post hoc* multiple comparisons (Dunn's test) via software SPSS 16.0 (IBM corporation, Armonk, USA). A value of *P* < 0.05 was considered to be significant.

## 3. Results

### 3.1. The Hypoglycaemic Effect of LBPS in Diet-STZ-Induced Diabetic Rats

**Figure 1:**
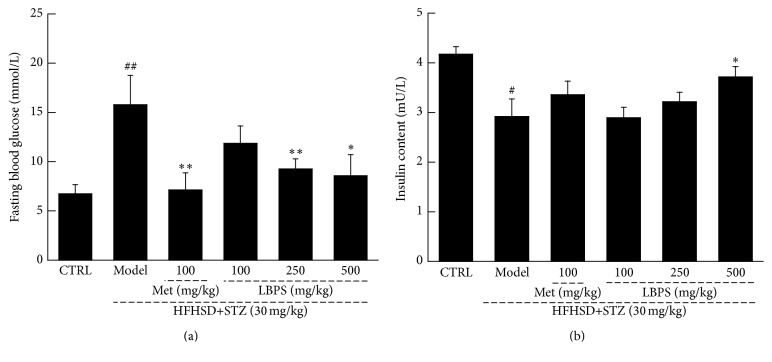
Compared with diabetic rats, four-week 100 mg/kg of metformin (Met) and* Lycium barbarum* polysaccharide (LBPS) at doses of 100, 250, and 500 mg/kg positively regulated the abnormal changes on (a) fasting plasma glucose level and (b) serum insulin concentration. Data are expressed as mean ± SD (*n* = 8) and analyzed by using a one-way ANOVA. ^#^ *P* < 0.05 and ^##^ *P* < 0.01 versus normal controls; ^*∗*^ *P* < 0.05 and ^*∗∗*^ *P* < 0.01 versus diabetic model rats.

Compared with normal controls, the enhanced water and food intake (*P* < 0.05; Table 1), blood glucose levels (*P* < 0.01; Figure 1(a)), and the inhibited growth rate (*P* < 0.05; Table 1) were observed after STZ injection in diabetic rats. Four-week Met (100 mg/kg) treatment relieved the abnormal alternations (*P* < 0.05; Table 1 and Figure 1). Compared with nontreated diabetic rats, LBPS at 500 mg/kg resulted in 40.5% increment of bodyweight (*P* < 0.05; Table 1), 46.1% reduction of water intake (*P* < 0.05; Table 1), 49.8% reduction of food intake (*P* < 0.05; Table 1), and 45.6% reduction of fasting blood glucose levels (*P* < 0.05; Figure 1(a)). Furthermore, a 27.4% enhancement of serum insulin concentration was noted in 500 mg/kg of LBPS-treated diabetic rats compared with nontreated group (*P* < 0.05; Figure 1(b)).

**Figure 2:**
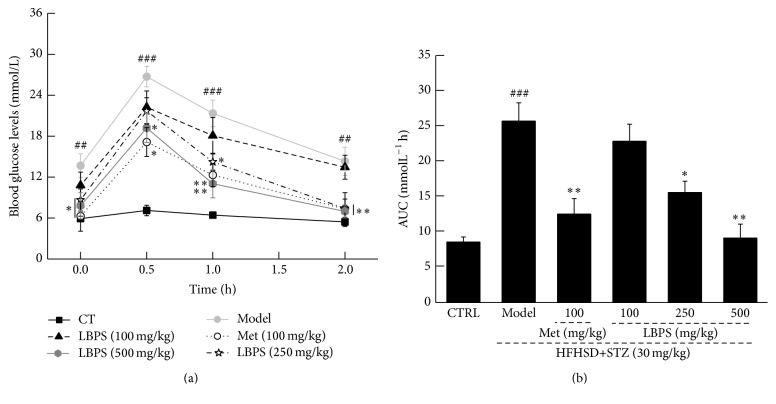
After the final treatment, all rats were orally treated with 2 g/kg of D-glucose; the changes of (a) plasma glucose and (b) area under the curve of glucose were detected. Data are expressed as mean ± SD (*n* = 8). ^##^ *P* < 0.01 versus normal controls; ^###^ *P* < 0.001 and ^*∗*^ *P* < 0.05 and ^*∗∗*^ *P* < 0.01 versus diabetic model rats.

Within 30 minutes of starting OGTT, nearly twofold blood glucose concentration was noted compared with its initial control value. Met and LBPS prevented blood glucose levels from shooting up significantly from 30 min to 120 min (*P* < 0.05, Figure 2(a)). The suppressive effects of Met and LBPS on AUC further confirmed their hypoglycaemic activities (*P* < 0.05, Figure 2(b)).

### 3.2. The Antioxidative Effects of LBPS in Diabetic Rats

**Figure 3:**
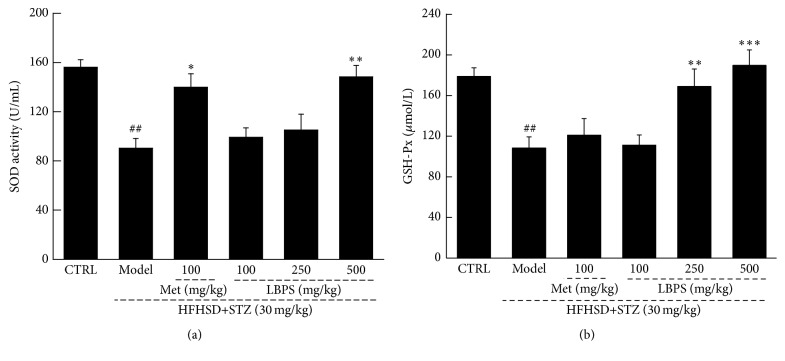
Met and LBPS enhanced the activities of (a) SOD and (b) GSH-Px in serum after four-week treatment in diabetic rats. Data are expressed as mean ± SD (*n* = 8) and analyzed by using a one-way ANOVA. ^##^ *P* < 0.01 versus normal controls; ^*∗*^ *P* < 0.05, ^*∗∗*^ *P* < 0.01, and ^*∗∗∗*^ *P* < 0.001 versus model rats.

Oxidative stress has been implicated in inflammation which is directly related to the activities of SOD and GSH [19]. Low serum concentrations of SOD and GSH-Px were noted in nontreated diabetic rats (*P* < 0.05; Figure 3). LBPS at dose of 500 mg/kg enhanced 39.2% of SOD level (*P* < 0.01; Figure 3(a)) and 75.1% of GSH-Px level (*P* < 0.001; Figure 3(b)) in serum of diet-STZ-induced diabetic rats compared with model group. Interestingly, Met only enhanced 55.3% SOD serum level in diabetic rats (*P* < 0.05; Figure 3(a)) but failed to regulate GSH-Px levels (Figure 3(b)).

### 3.3. The Effect of LBPS on Renal Function in Diabetic Rats

**Figure 4:**
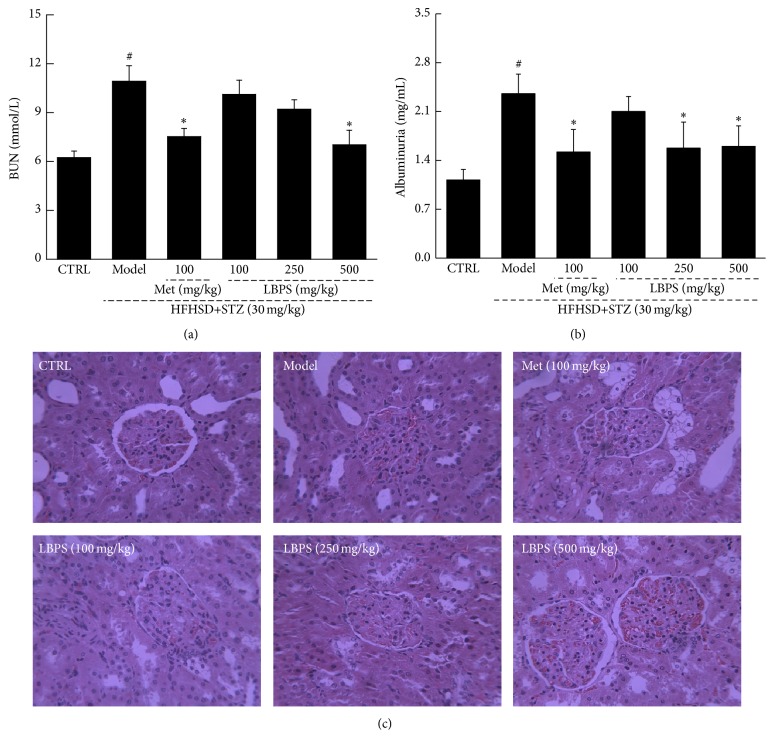
Diet-STZ-induced diabetic rats were orally treated with or without Met and LBPS at indicated doses for four weeks. The levels of (a) BUN and (b) urine albuminuria were detected. (c) Histopathological changes in kidney were analyzed through H&E staining (*n* = 6; ×400). Data are expressed as mean ± SD (*n* = 8) and analyzed by using a one-way ANOVA. ^#^ *P* < 0.05 versus control; ^*∗*^ *P* < 0.05 versus model rats.

BUN is one of the important factors renal dysfunction manifests. Met and LBPS significantly reduced the hyperlevel of BUN in diabetic rats (*P* < 0.05; Figure 4(a)). Albuminuria, considered as the hallmark for diabetic nephropathy [20], strikingly increased in diabetic rats (*P* < 0.01; Figure 4(b)). Both Met and LBPS strikingly reduced the albuminuria levels after four-week treatment in diabetic rats (*P* < 0.05; Figure 4(b)). In diabetic rats, glomerular basement membrane thickening or mesangial proliferation and inflammatory infiltrate injuries were observed, which were strongly ameliorated by Met and LBPS (Figure 4(c)).

### 3.4. The Anti-Inflammatory Effects of PBPS in Diabetic Rats

**Figure 5:**
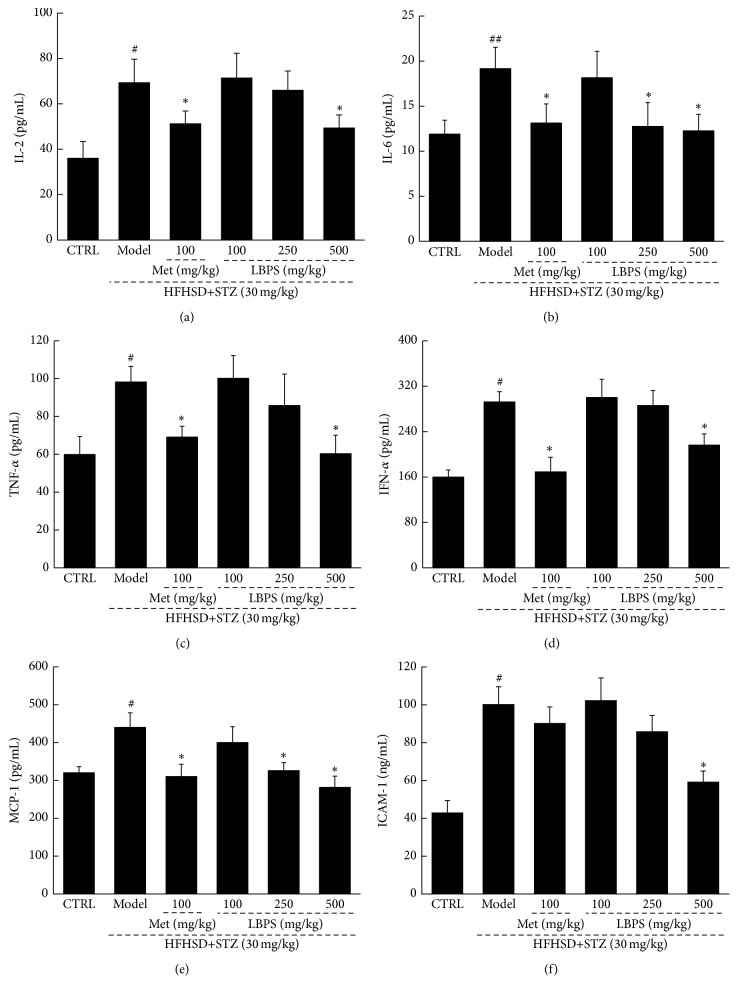
Diet-STZ-induced diabetic rats were orally treated with 100 mg/kg of Met and LBPS at doses of 100, 250, and 500 mg/kg for four weeks. The effects on the serum levels of (a) IL-2, (b) IL-6, (c) TNF-*α*, (d) IFN-*α*, (e) MCP-1, and (f) ICAM-1 were analyzed via ELISA method. Data are expressed as mean ± SD (*n* = 8) and analyzed by using a one-way ANOVA. ^#^ *P* < 0.05 and ^##^ *P* < 0.01 versus normal controls; ^*∗*^ *P* < 0.05 versus diabetic model rats.

Hyperglycemia leads to kidney damage associated with the severe inflammation characterized by the release of multiple inflammatory factors. Diabetic rats have exhibited the significant increase in serum levels of IL-2, IL-6, TNF-*α*, IFN-*α*, MCP-1, and ICAM-1 compared with normal rats (*P* < 0.05; Figure 5). Except for ICAM-1 level, Met reduced these hyperlevels of inflammatory factors (*P* < 0.05; Figure 5). LBPS showed similar effects on inflammatory cytokines regulation. Compared with diabetic rats, LBPS at 500 mg/kg caused a significant reduction in the serum levels of IL-2 (28.4% reduction; *P* < 0.05; Figure 5(a)), IL-6 (35.9% reduction; *P* < 0.05; Figure 5(b)), TNF-*α* (38.8% reduction; *P* < 0.05; Figure 5(c)), IFN-*α* (34.8% reduction; *P* < 0.05; Figure 5(d)), MCP-1 (36.1% reduction; *P* < 0.05; Figure 5(e)), and ICAM-1 (40.9% reduction; *P* < 0.05; Figure 5(f)).

### 3.5. LBPS Regulated the Activation of NF-*κ*B

**Figure 6:**
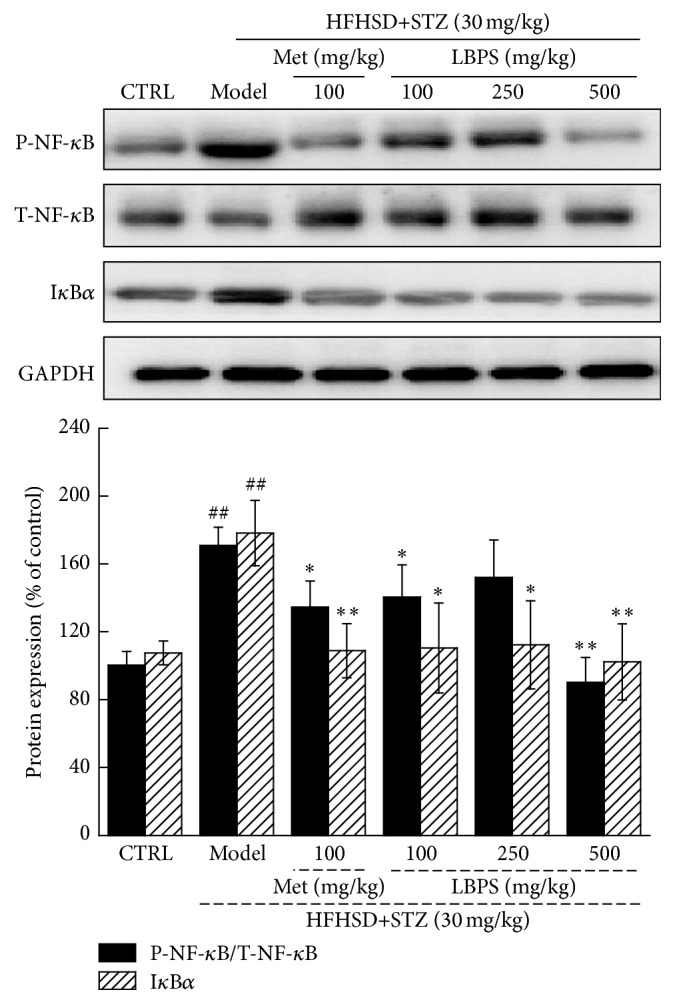
After four-week Met and LBPS treatment, the expression of P-NF-*κ*B, T-NF-*κ*B, and I*κ*B*α* in kidney was determined using western blot. Quantification data of the expressions of P-NF-*κ*B and I*κ*B*α* was normalized by T-NF-*κ*B and GAPDH, respectively. Data are expressed as mean ± SD (*n* = 4) and analyzed by using a one-way ANOVA. ^##^ *P* < 0.01 versus normal controls; ^*∗*^ *P* < 0.05 and ^*∗∗*^ *P* < 0.01 versus diabetic model rats.

The activation of NF-*κ*B p65 is controlled via the inhibitory protein I*κ*B*α*, which is considered to regulate multiple inflammatory factors. The strikingly enhanced expression of I*κ*B*α* and activities of NF-*κ*B were observed in the kidney tissues of diet-STZ-induced diabetic rats (*P* < 0.01; Figure 5). Compared with model group, both Met and LBPS significantly restored these expressions to normal levels (*P* < 0.05; Figure 6).

## 4. Discussion

Diet-STZ-induced diabetic rat model was successfully established for analysis of LBPS-mediated antidiabetic and antinephritic effects. Compared with diabetes model rats, the improved weight growth and reduced fasting blood glucose level were observed in LBPS-treated rats which indicates its hyperglycemic activity. Due to the more sensitivity of OGTT in glucose disregulation compared to that of fasting plasma glucose [1], the antidiabetic effect of LBPS was verified via OGTT. Hyperglycaemia decreased insulin secretion which is associated with the reduction in *β*-cell mass and impaired *β*-cell function [21]. LBPS strongly enhanced insulin secretion in diabetic rats and it indicates the protective activities of LBPS on *β*-cell damages in hyperglycaemia.

Oxidative stress is recognized as a major cause of diabetic complications. The disequilibrium of physical function in diabetic patients causes accumulation of reactive oxygen species (ROS); meanwhile, SOD and GSH-Px are primary enzymes for elimination of ROS [22]. SOD catalyzes the conversion of superoxide into hydrogen peroxide and oxygen, and GSH-Px scavenges the hydroxyl radical [23]. High blood glucose level leads to the autooxidative glycosylation of proteins [24], which further causes tissue damage. Antioxidant compounds have been recognized as an effective way to rescue the destruction of pancreatic beta-cell caused by alloxan [25].* Cordyceps militaris* presents excellent antidiabetic and antinephropathic activities via modulating the oxidative system including the enhancement of SOD and GSH-Px levels [18]. LBPS-mediated hyperglycemic activity may be related to the normalization of oxidative system.

Interestingly, oxidative stress in diabetics favors the imbalance of endothelial function, and this is an important step in inflammation [26]. During diabetic nephropathy, the apoptosis of podocyte is controlled by oxidative stress [27]. It has been confirmed that inflammatory and immunosuppressive factors are involved in hyperglycemia which causes glomerular hypertrophy and glomerular basement membrane thickening [28]. The overexpression of IL-2 enhanced the expression of endogenous cytokines, such as INF-g [29], which may be responsible for deteriorating glomerular damages [30]. IL-6 and TNF-*α* elevation contributed to cell damage and influenced *β*-cell development [31]. LBPS not only normalized L-2, IL-6, TNF-*α*, and IFN-*α* in serum, but also enhanced the serum concentration of ICAM-1 and MCP-1 in diabetic rats. In clinical trials, inflammatory stimuli have caused the disorder expressions of adhesion molecules and chemokines, especially in nephritis patients [32]. Via regulating inflammatory factors, especially for ICAM-1, and lowering uric acid, tubulointerstitial injury is reduced in diabetes [33]. All these data suggest that LBPS possesses antidiabetic nephropathy in diet-STZ-induced rat model via regulating inflammatory factors which may be controlled by oxidative stress. However, more experiments will be performed to find the detail connection between oxidative stress and inflammatory cytokines.

Furthermore, LBPS reduces the expression of phosphor-NF-*κ*B and I*κ*B*α* in kidney of diabetic rats. NF-*κ*B, a nuclear transcription factor, regulates various genes related to inflammation and autoimmune diseases [34]. NF-*κ*B displays the central role in renal protection via modulating proinflammatory cytokine [35]. Overexpression of NF-*κ*B in glomerular cells is responsible for nephritis in rats [36], which is reported to be controlled by protein I*κ*B*α*. Previous study also proves that the activation of NF-*κ*B is involved in cobalt chloride-mediated oxidative stress and inflammation in human renal proximal tubular epithelial cells [37]. Via modulation of NF-*κ*B,* Cordyceps militaris* fruit body extracts ameliorate cationic bovine serum albumin-induced membranous glomerulonephritis by attenuating oxidative stress and renal inflammation [38]. I*κ*B inhibitor, BAY 11-7082, attenuates hyperglycemia-mediated oxidative stress and renal inflammation in diabetic rat models via downregulating NF-*κ*B activation [39]. Altogether, NF-*κ*B contributes to LBPS-mediated anti-inflammatory and antioxidant properties in diabetes rat models.

In conclusion, via inhibiting the activation of NF-*κ*B,* Lycium barbarum* polysaccharide has attenuated oxidative stress and inflammation to show antidiabetic and antinephritic effects in diet-STZ-induced diabetic rats.* Lycium barbarum* can be a potential Chinese traditional medicine for diabetes mellitus and nephritis complications treatment.

## Figures and Tables

**Table 1:** Results on the body weight gain, food intake, water intake, and urine excretion in each experimental group.

Group	Dose (mg/kg/d)	Bodyweight gain/g	Water intake/mL	Food intake/g
CTRL	—	6.58 ± 1.27	45.00 ± 9.68	10.72 ± 2.60
Model	—	6.69 ± 2.09	89.00 ± 6.42^b^	19.85 ± 1.55^b^
DH-treated	100	30.06 ± 1.87^a^	43.52 ± 7.91^a^	8.97 ± 2.19^a^
LBPS	100	5.87 ± 2.32	94.17 ± 12.31	16.33 ± 2.50
	250	23.13 ± 1.81^a^	67.00 ± 13.11	11.41 ± 1.54^a^
	500	28.67 ± 3.16^a^	47.78 ± 8.14^a^	9.97 ± 1.89^a^

Data are expressed as mean ± SD (*n* = 8/group) and analyzed using ANOVA followed by Dunn's test.

^a^Statistical significance compared with diabetic model rats (*P* < 0.05).

^b^Statistical significance compared with control rats (*P* < 0.05).
